# Clinical pharmacist interventions in ambulatory psychogeriatric patients with excessive polypharmacy

**DOI:** 10.1038/s41598-022-15657-x

**Published:** 2022-07-06

**Authors:** Matej Stuhec, Kaja Zorjan

**Affiliations:** 1 Department of Pharmacology, Faculty of Medicine Maribor, University of Maribor, Maribor, Slovenia; 2 Department of Clinical Pharmacy, Ormoz Psychiatric Hospital, Ptujska Cesta 33, 2270 Ormoz, European Union Slovenia; 3 Faculty of Pharmacy, University of Ljubljana, Askerceva Cesta 7, 1000 Ljubljana, European Union Slovenia

**Keywords:** Health care, Medical research

## Abstract

Psychogeriatric primary care patients are frequently treated with excessive polypharmacy (≥ 10 medications), leading to complications and increased costs. Such cases are rarely included in treatment guidelines and randomized controlled trials. This paper evaluates the impact of clinical pharmacist medication reviews on the quality of pharmacotherapy in primary care psychogeriatric patients with excessive polypharmacy. The retrospective observational multicentric pre-post study included patients (aged 65 or above) treated with at least one psychotropic and ten or more medications. Clinical pharmacists’ recommendations were retrieved from medication review forms for the period 2012⁠–⁠2014. The study outcome measures were the number of medications, potentially inappropriate medications in the elderly (PIMs), potential drug-drug interactions which should be avoided (pXDDIs), and adherence to treatment guidelines. The study included 246 patients receiving 3294 medications, of which 14.6% were psychotropics. The clinical pharmacists proposed 374 interventions in psychopharmacotherapy. The general practitioners accepted 45.2% of them (169). Accepting clinical pharmacist recommendations reduced the total number of medications by 7.5% from 13.4 to 12.4 per patient (p < 0.05), the total number of prescribed PIMs by 21.8% from 312 to 244 (p < 0.05), the number of pXDDIs by 54.9% from 71 to 31 (p < 0.05) and also improved treatment guidelines adherence for antidepressants and antipsychotics (p < 0.05). Clinical pharmacist interventions significantly improved the quality of psychopharmacotherapy by reducing the total number of medications, PIMs, and pXDDIs. Accepting clinical pharmacist interventions led to better treatment guidelines adherence.

## Introduction

Polypharmacy is frequent in elderly patients with mental disorders and can result in adverse outcomes and treatment failures. Polypharmacy often leads to treatment complications, increased risk of side effects, and increased treatment costs^1^. A Swedish study (*N* = 630,743) reported a strong association between the number of prescribed drugs and potential drug-drug interactions (pDDIs)^2^. A 1990 study by Goldberg et al. found a 38% increase in pDDIs for patients with four medications and an 82% increase for patients with seven or more medications^3^. Psychiatric polypharmacy with two or more psychotropics is increasing in all age groups and is particularly critical in elderly patients, who often require medications for several conditions^4^. Elderly patients are thus particularly vulnerable and require careful medication selection.

Collaboration between physicians and clinical pharmacists (CPs) in treatment has been gaining momentum and demonstrated positive effects^5–7^. Research in recent decades focused on cardiovascular diseases in primary care settings and demonstrated a decrease in DDIs, adverse drug reactions, and treatment costs from involving pharmacists in the pharmacotherapy process^5–7^. A Swedish study showed that when the interventions of a CP were considered, the therapeutic effect improved in 68% of the patients, and adverse drug reactions were prevented in 32% of cases^5^. A German study reported an 80% decrease in the likelihood of drug-related problems following a CP’s inclusion into the treatment process^6^. A US study reported an improvement in medication safety from the inclusion of a CP in an interdisciplinary team of an intensive care unit^7^. A systematic review of 12 studies on patients with heart failure showed that pharmaceutical care (e.g., medication review, patient education, encouraging patient participation in drug treatment) significantly reduced hospitalizations but not mortality^8^.

Among elderly patients, mental disorders are common and highly comorbid with somatic diseases^9^. Severe depression occurs in 4.6–9.3% of the elderly, and the likelihood of developing depressive disorders increases significantly after the age of 75, which leads to the frequent use of psychotropics^9^. Depression most likely affects seniors with other chronic illnesses and cognitive impairments, where symptoms of helplessness and a loss of willpower are often present. The disease may exacerbate the clinical outcomes of associated diseases. There is a link between depression and Alzheimer's disease, diabetes, stroke, and pain^10–13^. Most anxiety disorders occur in the elderly simultaneously with depression or as one of its symptoms^14^. In an increasingly aging population, dementia is also rising with symptoms that include memory loss, difficulty thinking and communicating, and a reduced ability to perform daily activities. Although not a recommended long-term treatment, antipsychotics are often prescribed for dementia. Old age is also associated with shorter sleep times, frequent awakenings, and a decrease in sleep quality, which leads to a poorer quality of life^15^.

A survey of Slovenian retirement homes found that 73% of patients were prescribed at least one psychotropic and that 47% of patients received hypnotics or sedatives, 28% antipsychotics, 23% antidepressants, and 21% anxiolytics^16^. The frequency of prescribing psychotropic drugs in Slovenia is comparable to countries in Western Europe. Inappropriate use or inadequate dosing of these drugs in the elderly often leads to various adverse events and effects, such as fall-associated bone fractures, delirium, sedation, and cognitive impairment^17^. A study on the prevalence of potentially inappropriate medications (PIMs) prescribed to the elderly with a mental disorder found that at least 79% of all study participants had been prescribed at least one PIM. Of all PIMs, 70% were psychotropic drugs, most frequently antipsychotics and anxiolytics^18^. Appropriate interventions are needed to address irrational polypharmacy, PIMs, DDIs, and other medication-related problems in elderly patients with mental disorders. Such complex cases are frequent in clinical practice but not necessarily covered by existing treatment guidelines and randomized controlled trials. Consequently, observational studies in clinical settings are needed. This study aims to evaluate the impact of CP interventions on the quality of pharmacotherapy in primary care settings as measured by the total number of medications, pXDDIs, and PIMs, and adherence to treatment guidelines.

## Methods

### Study design and setting

This retrospective observational pre-post study was conducted in three primary health centers (Murska Sobota, Lendava, and Ljutomer) in NE Slovenia, providing primary care to approximately 100.000 inhabitants. We included patients aged 65 or above, who were receiving excessive polypharmacy (10 or more medications including at least one Anatomical Therapeutic Chemical Classification (ATC) code N psychotropic), had amental disorder diagnosis [defined by the 10th revision of the International Statistical Classification of Diseases and Related Health Problems (ICD-10)], and were referred to a CP by a GP for a medication review between 1.1.2012 to 31.12.2014^19^.

The medication review included the following essential aspects: potential and clinically significant DDIs, possible adverse events, existing drug indications, PIMs, and final recommendations. CPs communicated with GPs through a standardized medication review form and phone if necessary. GPs made the final decision to accept or reject the CP recommendations. See our other papers for further details on this service in Slovenia^20,21^.

### Inclusion, exclusion criteria, and data collection

The study included patients referred to a CP by their GP for a medication review, over which the researchers had no influence. Researchers only analyzed medication for chronic therapy or as-needed use, as other medications are not always included in medication reviews. Medications with two or more active substances were considered as two or more different medications. Medications with the same active substance in different doses were also considered as separate medications. Researchers categorized the CP recommendations into three intervention types (drug discontinuation, drug initiation, and dose adjustment) and excluded other possible interventions (e.g., food and drug administration timing). PIMs were determined with the potentially inappropriate medications in the elderly (PRISCUS) list (2010 version)^22^. The study only included potential pXDDIs, as defined by Lexicomp® 3.0.2. Adherence to treatment guidelines was assessed using guidelines and recommendations for specific conditions and (if necessary) summaries of product characteristics^23,24^.

The researchers had no direct contact with patients or GPs, as this was a retrospective study of patient charts. All retrieved patient data were anonymized. The patients’ baseline characteristics were recorded at the visit to the GP before the medication review. Changes in psychotropic pharmacotherapy were recorded at the patients’ next visit to a GP.

The research protocol was approved by the Slovenian National Medical Ethics Committee (Decision No. 0120-528/2016-2 KME 26/10/16, date: 10.11.2016) and the directors of the included health centers, and the director of the Health Insurance Institute of Slovenia. Informed consent from patients was not necessary because of the retrospective study design. All methods were performed in accordance with the relevant guidelines and regulations. The strengthening the reporting of observational studies in epidemiology (STROBE) checklist was used to ensure all required items of observational studies were included^24^.

### Study outcomes

The primary outcomes of this study were: (1) change in the number of medications per patient, (2) mean difference in pXDDIs, and (3) mean difference in PIMs. The secondary outcomes were treatment guidelines adherence for antipsychotics and antidepressants. Long-term clinical outcomes were not measured.

### Data analysis

Descriptive statistics represented baseline patient characteristics. The Shapiro–Wilk test was used for normality testing. A Wilcoxon signed-rank test was used to compare the data before and after medication reviews. Logistic regression was conducted to investigate the impact of patient age, gender, and the acceptance of CP recommendations on adherence to treatment guidelines for antidepressants and antipsychotics. A backward elimination method removed the independent variables that did not have a confounding effect. The significance level was set at *p* = 0.05. Patients with any missing data were excluded from the study. The data were processed in SPSS Statistics 25.0 for Windows. The research protocol was approved by the Slovenian National Medical Ethics Committee (Decision No. 0120-528/2016-2 KME 26/10/16, date: 10.11.2016) and the directors of the included health centers, and the director of the Health Insurance Institute of Slovenia. Informed consent from patients was not necessary because of the retrospective study design.

## Results

### General data (before interventions)

In this study, 34 patients were excluded because of missing data, and 246 patients were included, 168 women (68.3%) and 78 men (31.7%). The average patient age was 79.3 years (*Mdn* = 79, range 65–96). The average number of medications per patient was 13.4 (*Mdn* = 13). The highest number of prescribed medications per patient was 24. Psychotropics represented 14.6% of 3294 prescribed medications. PIMs represented 9.5% (312) of all prescribed medications, and 77.6% (191) of included patients were prescribed at least one PIM. Most PIMs (69.2%) were in the ATC group N, and benzodiazepines were the most frequently prescribed medication group.

**Figure 1:**
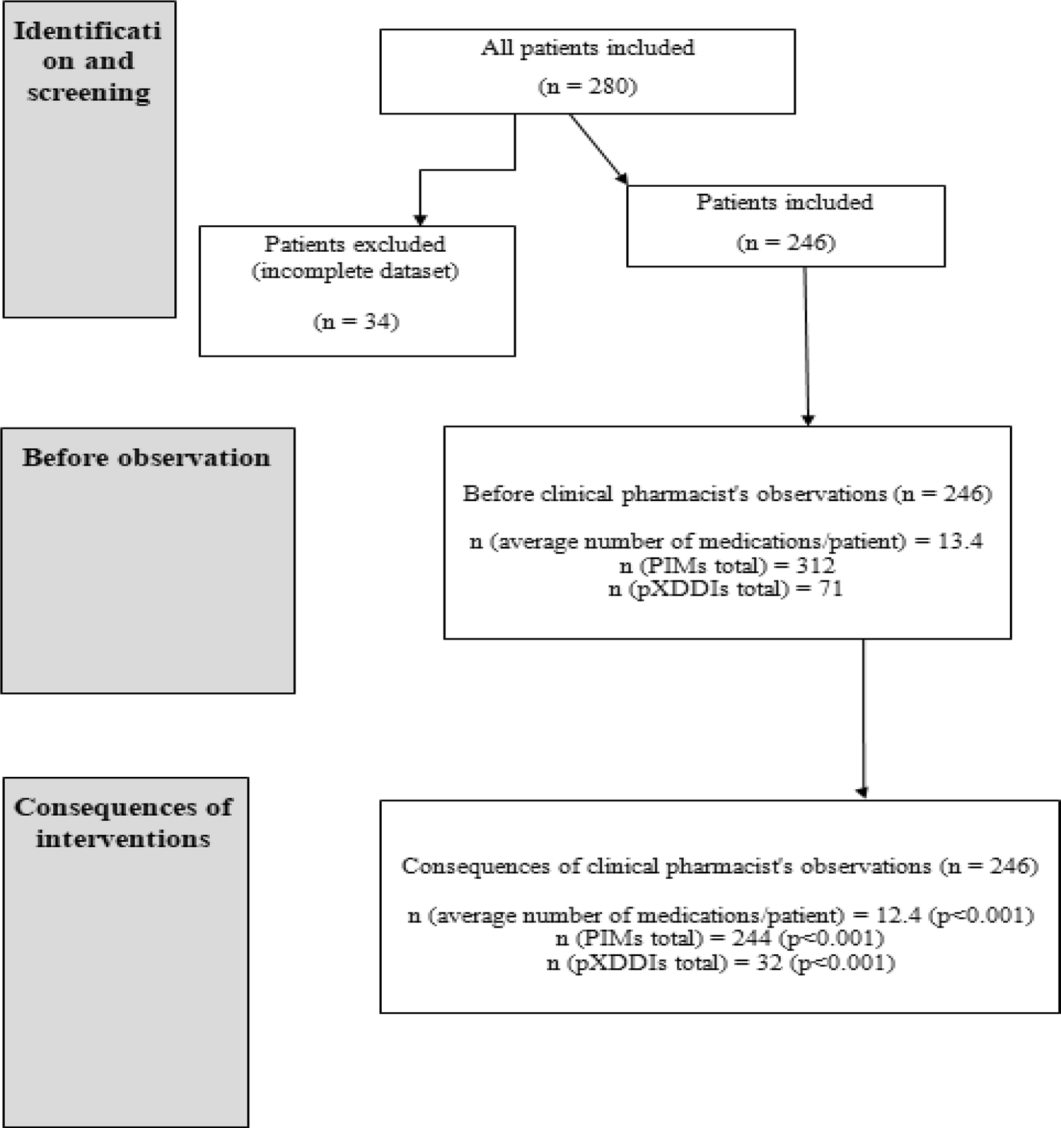
Flowchart.

Figure 1 shows the study’s flowchart (n = 246).

The antidepressants amitriptyline and maprotiline were prescribed in six and four cases, respectively. The antipsychotics haloperidol (> 2 mg daily), clozapine, olanzapine (> 10 mg daily), and fluphenazine were used in ten, six, five, and three patients respectively. At least one pXDDI was found in 21.1% (52) of patients, and there were 71 pXDDIs in total (*M* = 0.29 per patient).

### Primary outcomes

The study recorded 374 CP interventions (*M* = 1.52 per patient), most frequently drug discontinuations, followed by drug initiations and dose adjustments in 61.5%, 28.6%, and 10.9% of cases, respectively. See Table 1 for an overview of the intervention types across different groups of psychotropics. The GPs accepted 45.2% (169) of the interventions (Table 2). After the interventions, the mean number of medications per patient decreased from 13.4 to 12.4 per patient (*p* < 0.05), and the number of pXDDIs decreased from 71 to 32 (*p* < 0.05). The number of CP interventions and the proportion of interventions considered by the GP is shown in Table 2.

**Table 1:** Clinical pharmacist intervention types by psychotropic group (CP = clinical pharmacist).

	Antipsychotics	Antidepressants	Drugs for treating dementia	Drugs for treating insomnia	Benzodiazepines
Drug discontinuation	53	53	6	50	68
Drug initiation	20	30	8	23	26
Drug dose increase	1	7	3	0	0
Drug dose decrease	1	3	1	14	7
Together (% of all CP interventions)	75 (20.1%)	93 (24.9%)	18 (4.8%)	87 (23.3%)	101 (27.0%)

**Table 2:** Proposed and accepted clinical pharmacist interventions by psychotropic group.

	Antipsychotics	Antidepressants	Drugs for treating dementia	Drugs for treating insomnia	Benzodiazepines
Number of patients	87	113	45	105	112
Number of interventions (%)	75 (20.1%)	93 (24.9%)	18 (4.8%)	87 (23.3%)	101 (27.0%)
Accepted interventions (%)	24 (32.0%)	46 (49.5%)	13 (72.2%)	49 (56.3%)	37 (36.6%)

**Figure 2:**
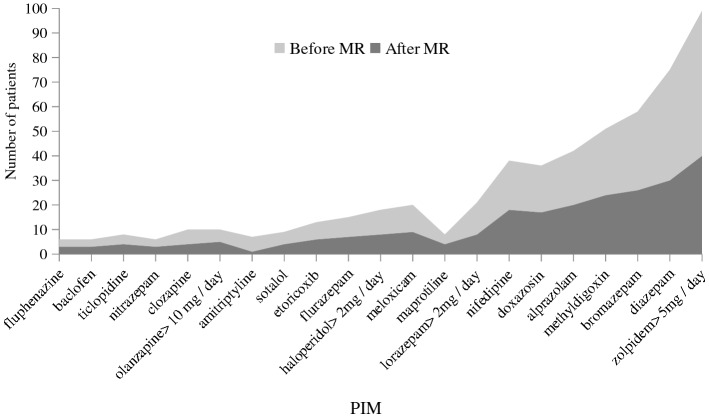
Number of Patients with PIMs Before and After MR (MR = medication review, PIM = potentially inappropriate medications in the elderly).

The number of proposed interventions was the highest for benzodiazepines (BZDs) (27% of all proposed interventions), but GPs accepted only 36.6% of them. The total number of PIMs decreased by 21.8% from 312 to 244 (*p* < 0.05). The proportion of PIMs in all prescribed drugs decreased from 9.5% to 7.4%. See Fig. 2 for the number of patients with PIMs before and after the CP interventions.

### Secondary outcomes

#### Treatment guidelines adherence

Treatment guidelines were not followed in 56 out of 113 patients treated with antidepressants (using the *Practice guidelines for treating patients with major depressive disorder*)^23^. Of all patients treated with antidepressants, 49.6% were not treated in accordance with treatment guidelines. That number decreased to 24.8% following the medication reviews. Treatment guidelines were not followed in 50.6% of patients treated with at least one antipsychotic (using *Guidance on the use of antipsychotics guidelines*)^24^. That number decreased to 35.7% after the GPs accepted 32.0% of the proposed interventions. See Table 3 for the results of treatment guidelines adherence.

**Table 3:** Treatment guidelines adherence for antidepressants and antipsychotics (*CP* clinical pharmacist, *MR* medication review).

	Antipsychotics	Antidepressants
Number of patients	87	113
Number of CPs interventions (%)	75 (20.1%)	93 (24.9%)
Number of CPs interventions accepted by GP (%)	24 (32.0%)	46 (49.5%)
Treatment guidelines adherence before MR (%)	49.4%	50.4%
Treatment guidelines adherence after MR (%)	64.3%	75.2%
Difference between treatment guidelines adherence before and after MR (%)	+ 14.9%	+ 24.8%

#### Regression model for guidelines adherence for antidepressants and antipsychotics

Logistic regression was performed to examine the effects of gender, age, and CP interventions' acceptance on adherence to treatment guidelines for antidepressants and antipsychotics. The models were statistically significant for both antidepressants (*χ*^2^ = 61.645; *df* = 5; *p* < 0.05) and antipsychotics (*χ*^2^ = 70,609; *df* = 5; *p* < 0.05). In both models, the acceptance of CP interventions was a significant predictor for treatment guidelines adherence (*p* < 0.05). Other independent variables did not have a significant impact. See Tables 4 and 5 for further details on the models for antidepressants and antipsychotics, respectively.

**Table 4:** Logistic regression analysis of treatment guidelines adherence for antidepressants.

	B-value	Wald	P-value	Exp (B)
Sex	0.634	0.668	0.414	1.886
Age	0.060	1.772	0.183	1.062
Acceptance of CP interventions (%)	− 0.061	12.425	0.000	0.941
Constant	− 0.419	0.012	0.914	0.658

**Table 5:** Logistic regression analysis of treatment guidelines adherence for antipsychotics.

	B-value	Wald	P-value	Exp (B)
Sex	− 0.604	0.403	0.526	0.547
Age	− 0.079	2.224	0.136	0.924
Acceptance of CP interventions (%)	− 0.043	8.550	0.003	0.958
Constant	8.705	3.345	0.067	6030.260

## Discussion

This study examined the collaboration between CPs and GPs in primary care settings with benefits for psychogeriatric patients treated with excessive polypharmacy.

The first significant result is that our study's acceptance rate of CP recommendations was lower than those of the same service in hospital settings. The acceptance rate by GPs in our study was 45.2%, which is comparable to 56.6% and 55% in a Belgian and Slovenian study, respectively^20,26^, but is much lower than 92.4% reported in a US study of a hospital setting^27^ This difference across settings could be explained by the fact that CPs are included in hospital ward teams, which is not the case in ambulatory settings. Another reason for low acceptance rate can be associated with different CPs roles in USA and Slovenia. In Slovenia, CPs have not the prescribing rights (e.g., dependent prescribing), which would improve acceptance rate and entire collaboration (e.g., collaborative practice agreement in the USA)^27^. Another reason is an independent prescribing practice tested in two primary care centres in Scotland. Clinical pharmacists’ interventions improved clinical outcomes in patients with depression and general anxiety disorders (GAD). Of the 75 patients, two-thirds (n = 47, 62.7%) were referred with mixed depression and anxiety diagnoses. There were 324 consultations (median 3, range 1–14) and 181 prescribing actions. At pilot completion, 34 patients (45.3%) had reduced PHQ-9 and/or GAD-7 scores by 50%. Patient questionnaires and interviews generated positive responses^28^.

In our other study, which includes only one primary care setting and 48 patients, the acceptance rate was 55%. This was conducted from 2015 to 2017 and had many important limitations (e.g., monocentric study and small sample size). The authors also did not check the impacts on adherence to treatment guidelines, which is a case in this study^21^.

The second important finding of this study is that medication reviews led to significant decreases in the average number of medications per patient and the total number of PIMs and pXDDIs, all of which are risk factors for polypharmacy. The decrease is consistent with previous studies. A randomized, double-blind controlled study in a Slovenian hospital setting reported a significantly lower number of pDDIs in the intervention group (*p* = 0.0034)^29^. A study of elderly patients with polypharmacy and cardiovascular diseases (*N* = 243) reported a 7.3%, 26.6%, and 47.8% decrease in the number of total medications, PIMs, and pXDDIs, respectively^30^. A US study of a medication review service found a significant 16.7% decrease in PIMs^31^, comparable to the 21.8% decrease in our study.

We found that psychotropics were often PIMs. The most frequent PIM was zolpidem dosed at over 5 mg daily, which was prescribed to 24% of patients in the study. It is a short-acting hypnotic similar to benzodiazepines but is structurally different. Doses ≤ 5 mg daily are recommended in the elderly as an alternative to long-acting BZDs because of a better safety profile and fewer side effects^22,35^. BZDs have a wide range of indications but can lead to increased sedation, hallucinations, cognitive impairment, increased risk of falls, and depression. They can be replaced with short-acting benzodiazepines (e.g., lorazepam at < 2 mg daily, zolpidem at < 5 mg daily), or antidepressants with a sedative effect such as mirtazapine and trazodone^22^. The frequent prescribing of BZDs in this study is consistent with our previous study on patients with mental disorders^20^. The reasons for receiving benzodiazepines listed in our study were restlessness, insomnia, anxiety, delirium, depressive disorders, and back pain. The exact reasons for receiving BZDs were not listed for some patients. The acceptance of CP recommendations on BZDs was relatively low (36.6%), which may be explained that patients have been prescribed as-needed BZD therapy for a long time. The documentation of some patients even stated that the physicians did not discontinue a particular BZD because the patient did not want to stop taking it. CP interventions reduced BZDs and zolpidem use which is in line with our previous studies^20^. Antipsychotics were also frequent PIMs in this study. Haloperidol and fluphenazine are classic antipsychotics, and the PRISCUS list suggests replacing them with atypical antipsychotics with a higher risk–benefit ratio (e.g., risperidone)^22^. The medication review service in this study reduced antipsychotic use, especially of haloperidol which is not recommended in higher doses in elderly patients^22^.

The last important finding is that the CP interventions were significantly related to better treatment adherence for antidepressants and antipsychotics. Psychotropic drugs are commonly used outside of their approved indications, which is often unavoidable in clinical practice^32^. However, some drugs for mental disorders are prescribed to patients for indications not in line with treatment guidelines: e.g., quetiapine as a hypnotic for the treatment of insomnia, which was also frequent in our study^33^. The improvement in treatment guidelines adherence is in line with our previous study of a single primary care setting (*N* = 49) that found the acceptance of CP recommendations (but not patient age) were significantly associated with improved adherence to treatment guidelines for antipsychotics (*p* = 0.041)^34^. Our study also observed significant improvements in adherence to treatment guides for antidepressants. This is not surprising, as a large naturalistic US study (*n* = 9090) reported that depression treatment, when pursued, was adequate in only 41.9% of treated 12-month cases (95% CI 35.9–47.9)^35^.

This study also has some significant limitations. It is a retrospective and non-interventional study. It contains selection bias because the inclusion of patients was based on the decision of GPs to refer patients for a medication review. The selection bias is partly mitigated as all screened patients were included, and the researchers were not involved in the treatment. However, randomized, prospective, and interventional studies would yield more robust results. This study also did not measure long-term clinical outcomes. Future research would benefit from using validated clinical questionnaires (e.g., Hamilton Depression Rating Scale) and long-term follow-up (6 or 12 months). Evidence for positive long-term effects comes from a retrospective observational study by Stuhec and Lah that reported all changes in pharmacotherapy, but one (99.1%) was still maintained 6 months after the CP interventions^21^. Lastly, some of the patient documentation was incomplete, and GPs sometimes did not record if a CP recommendation was accepted or not.

The study examined the effects of clinical pharmacist medication reviews on pharmacotherapy in psychogeriatric patients in three primary care settings. The results show a significant decrease in the total number of medications, potentially inappropriate medications, potential type-X drug-drug interactions, and a significant improvement in the adherence to treatment guidelines for antipsychotics and antidepressants. Further research should include a randomized study on the effects of this service in primary care settings.

## Data Availability

The datasets used and/or analyzed during the current study available from the corresponding author on reasonable request.
